# Application of the Delphi Method in the Study of Depressive Disorder

**DOI:** 10.3389/fpsyt.2022.925610

**Published:** 2022-07-07

**Authors:** Hengjin Wu, Linjie Xu, Yu Zheng, Lei Shi, Liangfan Zhai, FengQuan Xu

**Affiliations:** ^1^Guang’anmen Hospital, China Academy of Chinese Medical Sciences, Beijing, China; ^2^Graduate School of Beijing University of Traditional Chinese Medicine, Beijing, China; ^3^Xiyuan Hospital, China Academy of Chinese Medical Sciences, Beijing, China

**Keywords:** depressive disorder, Delphi study, clinical research, indigenous, treatment

## Abstract

Depressive disorder is a common mental disorder that has a high prevalence and low visiting rate, which caused patients years lived with disability. Due to the complexity of the depressive disorder, the Delphi method is a better choice compared with other commonly used methods, which provides a new perspective for the prevention and treatment of depression. This article will summarize the clinical studies of depressive disorders using the Delphi method from four perspectives, and summarize the advantages and disadvantages of the Delphi method in depressive disorders research, arguing that the Delphi method can cross the gap between clinical research and clinical practice, and is a highly practical part of the research process.

## Introduction

Depressive disorder is a common mental disorder manifested with depressed mood, loss of interest, insomnia or drowsiness, fatigue, psychomotor retardation, poor concentration, and memory loss as the main manifestations (1). A study indicates that in countries around the world, the prevalence of depressive disorder ranged from 6% (Shenzhen, China) to 21% (France), and a close look at WHO World Mental Health (WMH) results shows that on average about half of the respondents have positive symptoms of depression (2). Among them, major depression is the top three causes of years lived with disability (YLDs) in 136 countries (3), but only 16.5% of patients with major depression have received adequate treatment (4). As the modern population still harbors misconceptions and fears about mental disorders, a study showed that more than 80% of people agree that professional help should be sought for mental illness, but less than 40% are likely to take action (5). Regarding psychiatric medications, the same is true that with 57% of respondents agreeing that medications should be taken regularly, more than 60% believed that they would be harmful. Depressive disorders are heterogeneous with complex etiology (6), and their regression and prognosis are also influenced by biological, psychological, and social factors of the patient, and there is some disagreement in clinical treatment (7).

There are many commonly used methods to study depressive disorder, such as observational research and experimental research. In observational studies, inferences are usually drawn from a sample, so the representativeness of the sample directly affects whether the inference is correct or not. In contrast to observational studies, experimental studies need to strictly follow the three principles of statistical “replication, control, and randomization,” including setting up a certain number of replicate observation samples, establishing a control group, and adopting randomized grouping or random sampling to ensure the balance between groups. They have a higher level of evidence than Delphi studies, but the findings are not compatible with the complex clinical environment and may be difficult to apply practically. The Delphi Study is a reliable prediction and evaluation method combined with quantitative and qualitative, which uses an anonymous survey of expert opinions, and after several rounds of feedback, communication, and discussion, the expert opinions eventually converge to produce an expert consensus on the study objectives (8). A comparison of the advantages and disadvantages of the three can be seen in Table 1. Patients with depressive disorders have poorer adherence and greater individual differences than other disorders, making observational and experimental research more difficult to perform and more difficult to obtain valid conclusions. Using the Delphi method to conduct research at this time can help bridge the gap between theory and reality and lead to more realistic conclusions. Other commonly used clinical research methods may be difficult to handle due to the complex and varied clinical characteristics of depressive disorder and its various personalized characteristics. However, the Delphi method is a structured group communication process that can make decisions through collective subjective judgment and then deal with expert consensus by statistically aggregating opinions. It can also be compared with other research methods to exclude subjective factors and draw more accurate conclusions. In recent years, more and more scholars have started to use the Delphi method to conduct multi-faceted research on depressive disorders, and this article intends to summarize and organize the relevant studies, so as to provide references and inspiration for clinical research.

**TABLE 1:** Advantages and disadvantages of the three methods.

Name	Observational research	Experimental researches	Delphi study
Sample size	A large patient sample size is required to exclude error, but large individual differences in clinical patients can also introduce error and bias.		A smaller number of experts (around 15) can also lead to valuable conclusions, and ultimately to a statistical unification of expert opinion.
Compliance with study subjects	Patients may have low adherence and may easily disengage.		Compliance of specialists is usually high.
Problems of medical theology	Certain theoretical problems create problems for research.		No theoretical problems.
Factors affecting results	More quantity	High quantity and complexity	Low quantity
The difficulty of research implementation	A little difficult	Difficult	Not very difficult
Evidence level	High	Higher	low
Matching with the real world	Not very high	Not very high	Higher

## Methods

We conducted a search using PubMed and Embase with the keywords Delphi study, Delphi technique, depressive disorder, and depression. Papers on the above keywords were then screened and selected According to our inclusion criteria and exclusion criteria, papers should use the Delphi method, or use research methods that include the Delphi method, and papers should study depressive disorder or study subjects that include or are related to depressive disorder. Any article that was not related to the Delphi method or did not study depressive disorders should be excluded.

## Results

Using the above criteria, we screened 72 papers. We did not find reviews of Delphi studies on depressive disorders. In terms of specific research content, 6 papers (8% of studies) produced clinical guidelines, consensus, or quality indicators for different types of depressive disorders, 3 papers (4% of studies) made recommendations for the prevention of depressive disorders, 5 papers (7% of studies) improved and validated relevant diagnostic scales, 5 papers (7% of studies) provided consensus on the care needs of patients with depressive disorders, and 5 papers (7% of studies) 5 papers (7% of studies) provided a consensus on the care needs of patients with depressive disorders, 5 papers (7% of studies) provided references on emergency care for different types and severity of depressive disorders, 2 papers (3% of studies) told patients with depressive disorders how to help themselves, 8 papers (11% of studies) developed an expert consensus on the use of multiple medications, Four papers (5% of studies) examined other therapies and tools that have been helpful in depressive disorders, Sixteen papers (22% of studies) focused on the co-morbidity of depressive disorders with other disorders and made recommendations, and 18 papers (25% of studies) analyzed and explored depressive disorders in different populations, including adolescents, the elderly, pregnant parents, and physician (medical student) groups. Findings from our review were summarized by indigenous studies of depressive disorders, diagnosis and differential diagnosis of depressive disorders, treatment process for depressive disorders, and prevention and prognosis of depression disorder.

### Localization of Depressive Disorders by Delphi Method

Currently, the more recognized definition and diagnostic criteria for depressive disorders are based on the 5th edition of the Diagnostic and Statistical Manual of Mental Disorders (DSM-5) compiled by the American Psychiatric Association, and most of the scales used in clinical practice are developed based on the DSM-5. However, the occurrence and development of depressive disorders are complex, and different social backgrounds, economic levels, and cultural folklore can have an impact on their clinical manifestations and prognosis, so indigenous studies of depressive disorders are significant (9–12). In contrast, indigenous studies are mostly difficult to quantify due to the low level of evidence of the conventional studies (7). Comparatively, the expert experience and consensus obtained from disease studies based on original assessment modalities through Delphi studies provide a novel exploration for clinical study and are helpful for indigenous studies of depressive disorders.

Psychological scales are the most important means of assessing depressive disorders (13), but their reliability and validity, and applicability to a certain population still need corresponding localization studies. At this point, Delphi studies can facilitate the process of localization of psychological scales. Loneliness is closely related to depressive disorders and is a risk factor for depressive disorders that can be intervened, affecting the development, progression, and prognosis of depressive disorders (13–16), which in turn affects the physical and mental health of older adults. However, the lack of a valid instrument to evaluate loneliness has hindered the prevention of depressive disorders in older adults to some extent. Instead of designing a new instrument, it would be better to have a common instrument in English and Chinese, such as a localized translation of the 6-item De Jong Gierveld Loneliness Scale, which would help to integrate data from different centers around the world for cross-cultural analysis. To develop and validate a Chinese version of the 6-item De Jong Gierveld Loneliness Scale to better assess the risk of depression among the elderly in China, Leung et al. (17) issued a Chinese version of the De Jong Gierveld Loneliness Scale to 103 Hong Kong elderly people aged 60 years or older to conduct a statistical analysis of the reliability and validity of the scale, while the Delphi group later determined the validity of the scale content. The results showed that the total loneliness score was significantly and positively correlated with the direct measure of loneliness (rpb = 0.71; *p* < 0.001), which is considered the gold standard for assessing loneliness, indicating that the Chinese version of the De Jong Gierveld Loneliness Scale has good reliability and validity The application of this scale will be beneficial for better prevention of depressive disorders in the elderly.

A new, culturally adapted scale could also be developed through a Delphi study. Xie et al. (18) concluded that depression among older adults in China is not given enough attention, so they used the Delphi method to develop a culturally appropriate scale to screen for depression in older adults in a non-psychiatric context, and tested it for diagnostic reliability and validity, both with high levels. Their results showed that for the Chinese society, the presence of appetite or weight changes, sleep disturbances, and somatic complaints were the top three most valuable symptoms for the diagnosis of depression in older adults.

The Delphi study may also assist in localizing treatment tools. For example, Hart et al. (19) developed a series of local culture-based guidelines through the Delphi study in order to maintain indigenous mental health in Australia. The findings suggest that physicians must be aware of relevant cultural factors in mental illness, such as cultural behaviors that may mimic symptoms of mental illness, the important role of family and community, and the need to facilitate supporting relationships. For example, concerning cultural factors, where patients may mimic cultural behaviors that are symptomatic of mental illness. These findings are consistent with other works of literature related to culturally based care for depressive disorders.

### Using Delphi Research to Clarify the Diagnosis and Differential Diagnosis of Depressive Disorder

The clinical manifestations of depression are varied, but in clinical diagnosis and treatment, patients’ poor insight into their disease may mislead doctors, making it difficult for doctors to make a quick and accurate diagnosis (20–22). Traditional scales, such as Minnesota Multiphasic Personality Inventory (MMPI) have some defects, such as the large number of questions and long time-consuming, while simple scales, such as SDS, are difficult to assess accurately the severity of depression (23), it is difficult for patients with a depressive disorder to obtain psychological scale efficiently and comprehensively. There are many scales used in the clinical diagnosis of depressive disorder, and the reliability and efficiency of different scales are also difficult to choose. Nabbe (24) conducted a Delphi study on the selection of clinical scales and concluded that the Hospital Anxiety and Depression Scale and the Hopkins Symptoms Checklist-25 (HSCL-25) are sufficiently valid and reliable clinical options.

There is also a clinical dilemma that is difficult to identify with bipolar disorder type 1. To assess the value of Bipolar Spectrum Diagnostic Scale(BSDS) in the differential diagnosis of unipolar and bipolar depression, Juan Pablo et al. used BSDS to evaluate patients with a depressive episode, and then patients are reassessed using the Structured Clinical Interview for DSM-IV Axis I Disorders. The SCID-I diagnosis was compared with the BSDS screening results to analyze the accuracy, sensitivity, and specificity of BSDS screening for bipolar disorders. Studies have shown that in patients with depressive episodes in Mexican, a cut-off value of 12 reached the most stable sensitivity and specificity, with predictive powers higher than 0.80, which is basically consistent with foreign studies (25–27). This result suggests that BSDS screening for patients with bipolar disorder in depressive episodes has a certain degree of accuracy and specificity, but its sensitivity is not ideal (28).

Corresponding, McIntyre et al. (29) have used the Delphi method to create a rapid mood screening tool to identify both unipolar and bipolar disorder, which can screen for not only manic symptoms but also bipolar disorder, it also includes risk factors for bipolar disorder 1 (for example, the age of onset of depression) to help clinicians reduce the risk of misdiagnosing bipolar disorder 1 as a depressive disorder. The sensitivity and specificity of the tool were 0.88 and 0.80, respectively. The positive and negative predictive values were 0.80 and 0.88, respectively. The accuracy of the tool was higher than that of the other mood disorders questionnaire, at the same time, the questionnaire options were reduced by more than 50%. The use of this tool effectively addresses the need for a more comprehensive assessment of bipolar disorder 1, helping to reduce misdiagnosis and improve treatment regimens.

There is a lack of consensus on certain subtypes of depressive disorders, which can hinder evaluation, testing, and treatment, and the Delphi method can be used to guide clinical practice. Freitas et al. (30) conducted a Delphi study aimed at reaching an expert consensus on the defining factors of paternal depression during pregnancy and postpartum. They believe that perinatal paternal depression is “depression that starts during pregnancy or a year after birth.” Many for “depressed mood, negative thinking, physical symptoms (weight loss, sleep problems), as well as pregnancy or postpartum about a year ‘masked male depression symptoms.” Found several symptoms specific to this group, including confusion about fatherhood/concern about fatherhood and reduced involvement in caregiving activities, experiencing “conflict between who you should be and who you really are,” feeling trapped in life, and grieving over the loss of your old life and relationships; Experts also point out that there is a lack of appropriate tools for assessing perinatal paternal depression in response to these symptoms, even with male-specific depression tools such as the Gotland Male Depression Scale, there is no consensus on its use in this particular population, which may be due to the unique experiences of the father during the pregnancy of the partner or after the birth of the child.

### Use the Delphi Method to Evaluate and Improve the Treatment Process for Depressive Disorders

The clinical use of antidepressants needs extra caution, its side effects are various (31). Antidepressants bring pain to patients, but they may also reduce patients’ compliance to medication, induce patients’ sense of shame and increase patients’ anxiety (32), and it takes some time for antidepressants to take effect, dosage selection also needs to be considered, after taking a certain degree of addiction, the withdrawal will also have a considerable degree of withdrawal reaction (31, 33, 34). Antidepressants tend to be expensive, and discretionary use can put a financial strain on the health care system and on patients, which can ultimately translate into emotional stress (35–37). In response to these dilemmas, several researchers have evaluated the clinical use and health economics of antidepressants and other therapies, providing detailed expert advice on clinical drug selection.

Delphi study provides expert advice on clinical drug use, for example, Lee et al. (38) conducted a Delphi study to build local consensus and guide doctors to use bupropion for different mental illnesses. The study ultimately yielded 11 consensuses, covers indications, contraindications, side effects, etc. It provides a valuable reference for clinical medication and also puts forward a more detailed application consensus. Besides treatment for major depressive disorder, bupropion is also indicated for seasonal affective disorder and is particularly useful for patients with anhedonia, reduced motivation, weight concern, and sexual dysfunction; Off-label uses of bupropion include smoking cessation, attention deficit hyperactivity disorder (ADHD), bipolar disorder, Parkinson’s disease, depression, and occasionally for some anxiety disorders. Similarly, Simonetti et al. (39) reached an expert consensus on the importance of mood stabilizers represented by lithium-ion preparations combined with antidepressants in the treatment of chronic bipolar disorder and suggested that for the manic/mixed phase, whether used alone or in combination with mood stabilizers, second-generation antidepressants may be effective in the short to medium term. In addition to advising on the clinical application of a drug, there are also experts comparing multiple drugs through the Delphi study. To select the most suitable antidepressant drugs for the elderly, Agüera-Ortiz (40) led an expert group to select 21 common antidepressant drugs and evaluate the operation of different strategies in terms of efficacy and safety in dealing with drug-resistant cases. Considering drug therapy, agomelatine is the most widely mentioned drug in terms of safety in comorbid conditions. In general, venlafaxine, sertraline, and vortioxetine are the most commonly recommended antidepressants.

However, even if a patient is fully on-target for a drug, there is still a chance that the drug will not work well (41, 42). Selective serotonin reuptake inhibitors (SSRIs) are often recommended as the antidepressant of choice (43), but 30–40% of patients do not respond adequately to the first prescribed antidepressant (44), this may be due to the patient’s genotype insensitive to SSRIs. Oestergaard et al. (45) conducted a Delphi study on whether 5-HTTLPR polymorphism screening should be used in clinical problems of depression, expert opinion pointed out the impact of 5-HTTLPR pre-detection on clinical outcomes, the introduction of 5-HTTLPR genotyping will lead to 33.8, 48.2, 57.8, and 65.1% of patients reaching remission at 1, 2, 3, and 6 months, respectively. It is determined that, in some special situations the test could be combined with 5-HTTLPR to better predict patient characteristics of depressive responses resistance.

While using the medicine, in addition to considering whether the drug is suitable for the patient, it is also necessary to consider the cost that the patient needs to bear. At this time, the Delphi method is also often used for pharmacoeconomic evaluation and drug cost-effectiveness research. Wade et al. (46) used the Delphi method to study the cost and benefit of escitalopram and citalopram in the treatment of major depressive disorder in the United Kingdom. Escitalopram had a higher overall response rate and first-line success rate, and the average treatment cost was 15.7% lower than citalopram. This conclusion is also supported by a study by Nuijten et al. (47) in the Netherlands, where the favorable clinical benefit of escitalopram resulted in positive health economic benefits. In addition, Le Pen et al. (48) evaluated the potential economic benefits of fluoxetine versus tricyclic antidepressants (TCAs) in the treatment of mild to moderate depression, and Lenox-Smith et al. (49) assessed the cost-effectiveness ratio of venlafaxine versus fluoxetine and amitriptyline for the treatment of major depressive disorder in the United Kingdom. Both studies have shown that although the development of new drugs is expensive, better efficacy can bring higher social value and more economic savings, so slightly higher treatment costs should not be a hindrance to the first-line choice for new drugs to treat depressive disorders.

In addition to antidepressants, adjuvant therapy with other medications can also help with depressive disorders. The International Society for Research in Nutritional Psychiatry (ISNPR) developed the first practice guidelines for the use of n-3 polyunsaturated fatty acids (PUFAs) in the treatment of major depressive disorder in 2019 (50), but there is a gap between evidence and consensus. To strengthen these guidelines and improve their clinical applicability, Guu et al. (51) synthesized the evidence and clinical experience previously obtained through the Delphi methodology, and based on the support of a large body of evidence, ultimately “N-3 PUFAs are one of the potential adjunctive therapies for MDD in adults” reached the highest consensus. In addition to drug treatment, adjuvant therapy such as exercise therapy and mindfulness therapy can also help patients with depressive disorders. And in the information age, it is also possible to use information and communication technology (ICT) to improve depression. Gros et al. (52) have used the Delphi method to explore ways to help patients with depressive disorders through ICT, such as improving patients’ mood through games, and online psychological counseling through VR. These methods are very positive for the improvement of patients’ conditions.

### Consensus Study on Improvement of Prevention and Prognosis of Depression Disorder

It is difficult to predict the prognosis of patients with depression (53, 54), as most patients often relapse within a few years or require lifelong maintenance medication if they relapse more than once (55). So how to determine and improve the prognosis of patients is also of great concern to researchers. Based on patients with insights on the disease (including the understanding of disease, symptoms, the demand for treatment, and social consequences of mental illness) is related to the prognosis of mood disorders (20–22), Olaya et al. (56) through the Delphi study design and apply a scale of insight for depression patients. They found a correlation between patients’ poorer insight and a higher number of previous hospitalizations, but not with patients’ demographic characteristics, including gender, age, or education.

Similarly, patients leaving the hospital do not mean the end of the diagnosis and treatment process. Compared with other diseases, patients with depression disorder need spiritual care, and it is also an important part of daily care and follow-up of patients during the diagnosis and treatment process. A Delphi study by Koekkoek et al. (57) concerned many difficulties in the care of chronic depressive disorders, such as the risk of relapse, dependence on treatment, and the sense of hopelessness caused by recurrent episodes. With another Delphi study undertaken, Palmer (58) agreed on 5 points about how primary care settings serve depressive disorders and related conditions: including listening, understanding, compassion, providing thorough and competent diagnosis and management, regularly following and monitoring patients’ condition, facilitating patient visits, and providing comprehensive treatment and tailored care according to individual needs.

The rehabilitation of patients with depressive disorder not only needs the efforts of medical staff and caregivers, but also patients’ efforts play a significant role. Several experts have conducted Delphi studies, offering patients a range of steps that they can take to improve their situation, such as recognizing and acknowledging depression, how to avoid self-harm and suicide attempts, how to seek help from the outside world, and separating advice on how to deal with psychological crises (59–62). Shin et al. (63) tested the effect of self-help intervention on depressive symptoms and sub-threshold depression by Delphi, which showed that lifestyle change and psychological methods were the best choices for depression patients, while health care products were the least effective. They also found that psychiatrists (82.6%) and the general population (67.2%) were more likely to prefer self-help methods than depressed patients (28.4%), perhaps because of patients’ pessimism that they would not benefit from self-help strategies. In terms of specific operations, Morgan et al. (64) proposed a series of sub-threshold depression self-help strategies, aim to help reduce the sub-threshold depression degree, improve the quality of life of the people, thus preventing depression, the concreting content includes regular sleep and exercise, do like things, avoiding excessively overworked, learning relaxation techniques, associating with positive people, etc.

## Discussion

Due to the diversity of social background of the patients with depressive disorder, the disunity of the evaluation scale, the difficulty of clinical diagnosis and differential diagnosis, and the uncertainty of prognosis, the Delphi method is regarded as a better research method. A combination of the Delphi method was applied to study the localization of depressive disorder, diagnosis and differential diagnosis, treatment, and prognosis of the status, we found that in different regions, different cultural backgrounds, different economic characteristics of the social system, the application of each kind of different depression scale for assessment of all need to take into account the application of the reliability and validity. Given the clinical confusion that it is difficult to distinguish single depression from bidirectional depression, the establishment of an evaluation scale with high reliability and efficiency is an urgent problem to be solved in the clinic. The selection of clinical antidepressants is characterized by the contention of a hundred schools of thought and is affected by the lack of development of biomarkers for depression. Years of network meta-analysis established Sertraline Hydrochloride in the treatment of depressive disorder status, but the different social backgrounds, economic, the age of the onset of depressive disorder lead to the treatment of depressive disorder need accuracy, so the Delphi method research can be integrated clinical expert clinical experience, improve the accuracy of clinical treatment; The recurrence and prognosis of depression are difficult to be solved clinically, and the choice of different treatment schemes also leads to the difficulty in estimating drug withdrawal and disease recurrence. Delphi method can gather the clinical experience of experts in the clinical field and make clinical treatment more grounded, which is difficult to be achieved by other research methods.

As an anonymous expert investigation method, the Delphi method has obvious advantages and disadvantages. Though it is difficult to measure the reliability and validity of Delphi method, and it is requires a long research period, obtains a low level of evidence, and difficult to reach an accurate consensus in the face of controversial content, Delphi research avoids the shortcoming of low credibility of general questionnaire survey through several rounds of expert consultation and makes comprehensive statistical analysis of the conclusions, and finally transforms the personal experience of experts into expert consensus on a certain aspect. Due to the complexity of depression, it is difficult for traditional research methods to obtain high-level evidence, and the research conclusions do not match the complex clinical environment, so it is difficult to apply to clinical practice. Delphi research can combine the existing research results with the rich experience of experts, which is a shortcut to the clinical application of research results. At the same time, in the process of continuous feedback and communication with experts, Delphi research is better at discovering the shortcomings of the current research, which can point out the direction of the next research. When we use the Delphi method in a suitable environment and time for research, it may bring more help to our research.

## Author Contributions

FX was responsible for the conception of the thesis. HW was responsible for writing the thesis. LX, YZ, LS, and LZ were responsible for thesis revision. All authors contributed to the article and approved the submitted version.

## Conflict of Interest

The authors declare that the research was conducted in the absence of any commercial or financial relationships that could be construed as a potential conflict of interest.

## Publisher’s Note

All claims expressed in this article are solely those of the authors and do not necessarily represent those of their affiliated organizations, or those of the publisher, the editors and the reviewers. Any product that may be evaluated in this article, or claim that may be made by its manufacturer, is not guaranteed or endorsed by the publisher.
